# Supplementary material for: S- and N-Co-Doped TiO2-Coated Al2O3 Hollow Fiber Membrane for Photocatalytic Degradation of Gaseous Ammonia
Source: Membranes (Basel). 2022 Nov 4;12(11):1101. doi: 10.3390/membranes12111101 (PMC9696333; doi:10.3390/membranes12111101)
Supplement: Supplementary file 1 [file membranes-12-01101-s001.zip › membranes-1972740-supplementary.pdf]

membranes

S- and N-codoped TiO_2-coated Al_2O_3 hollow fiber membrane for photocatalytic degradation of gaseous ammonia

Jae Yeon Hwang †, Edoardo Magnone †, Jeong In Lee, Xuelong Zhuang, Min Chang Shin and Jung Hoon Park *

[1] Department of Chemical and Biochemical Engineering, Dongguk University, 30, Pildong-ro 1 gil, Jung-gu, Seoul, 04620, South Korea
† These authors contributed equally to this work.
* Correspondence: Tel: +82-2-2260-8598. Fax: +82-2-2260-8729. E-mail address: pjhoon@dongguk.edu

Supplementary Materials

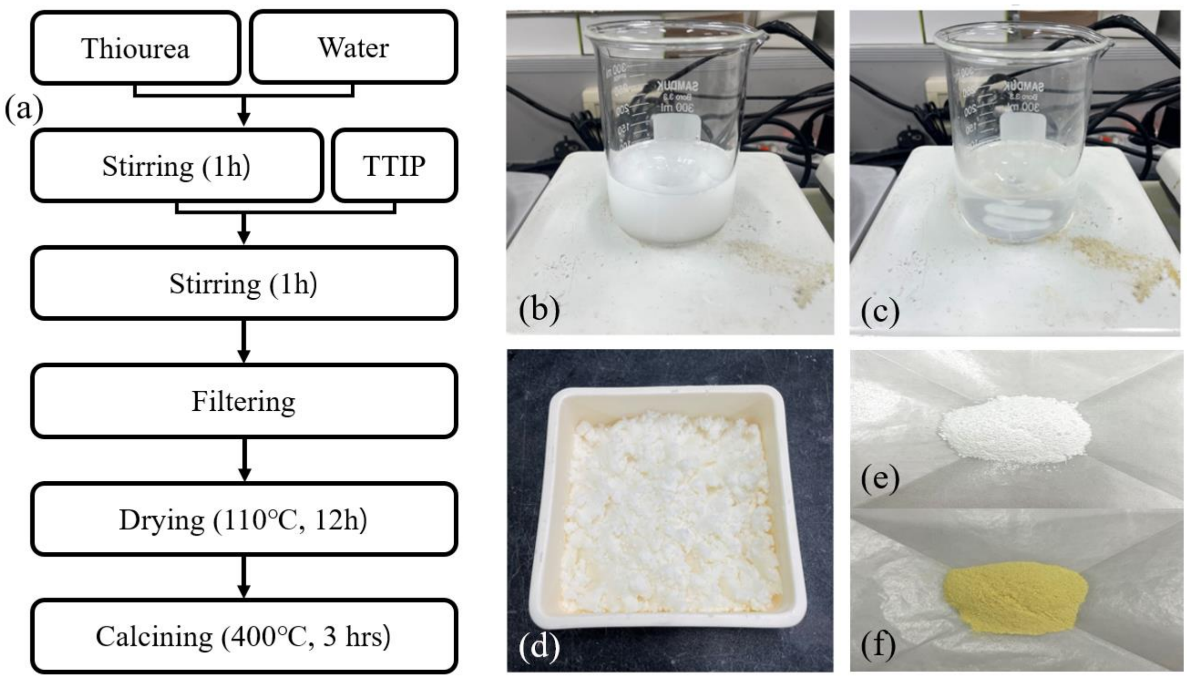

Figure S1. (a) Schematic procedure of undoped TiO_2 and S,N-doped TiO_2 synthesis (TTIP = Titanium (IV) isopropoxide). (b) Initial thiourea and deionized water solution. (c) Thiourea and deionized water solution stirred for 1 hour. (d) Filtered product. Digital photographs of the prepared (e) undoped TiO_2 and (b) S,N-doped TiO_2 powders after calcination process.

Table S1. Unit-cell parameters (a=b, c, and unit cell volume) for undoped TiO_2 and S,N-doped TiO_2 photocatalyst powders (Symmetry class: Tetragonal; Space group: I41/amd; Space group number: 141).

Sample	*a* (Å)	*b* (Å)	*Vol.* (Å^3)
Undoped TiO_2	3.7849	9.4883	135.92
Codoped S,N-TiO_2	3.7916	9.5055	136.65

Table S2. XPS elemental analysis of undoped TiO_2 and S,N-doped TiO_2 photocatalyst powders.

Sample	Elemental Amount (at.%)			
	Ti	*O*	*S*	*N*
Undoped TiO_2	26.4	73.6	-	-
Codoped S,N-TiO_2	25.1	70.1	3.2	1.6

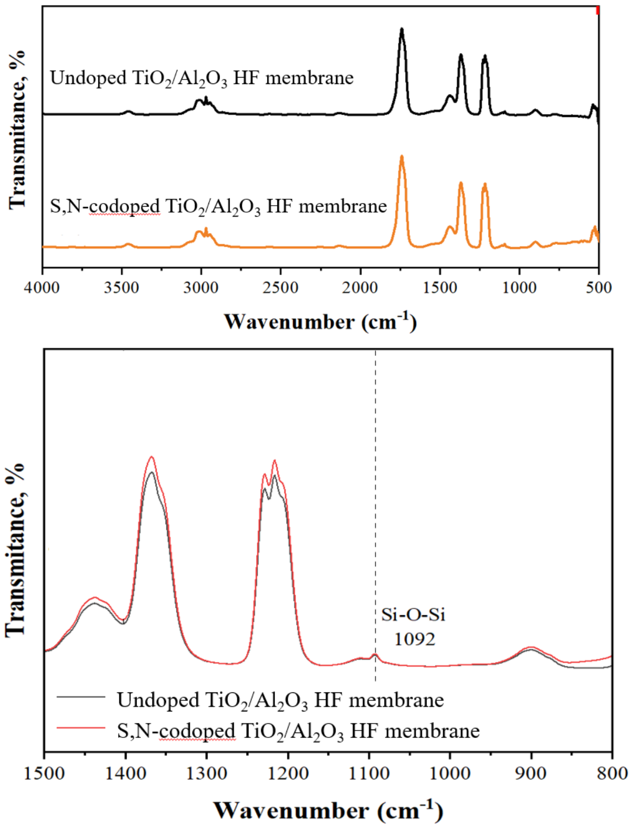

Figure S2. FT-IR spectra of undoped TiO_2 and S,N-doped TiO_2 photocatalyst powders.

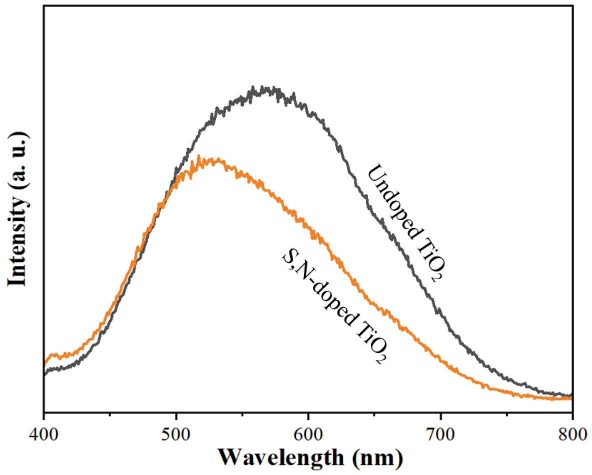

Figure S3. Photoluminescence spectra of undoped TiO_2 and S,N-doped TiO_2 photocatalyst powders.

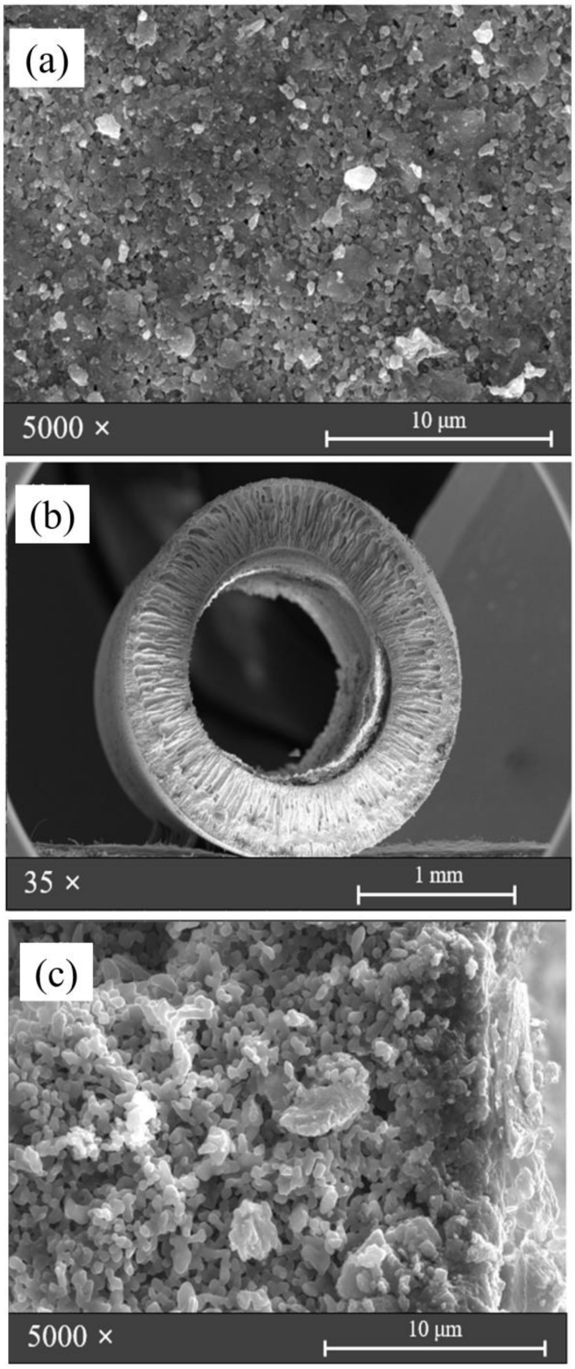

Figure S4. SEM images of the S,N-doped TiO_2/Al_2O_3 HF membrane: (a) surface of the S,N-doped TiO_2 film, and cross-sections with two different magnifications (b) 35X, and (d) 5000X.
